# Individual and contextual factors in the Swedish Nutrition Care Process Terminology implementation

**DOI:** 10.1177/18333583221133465

**Published:** 2022-11-21

**Authors:** Elin Lövestam, Ylva Orrevall, Anne-Marie Boström

**Affiliations:** 1 Department of Food Studies, Nutrition and Dietetics, 8097 Uppsala University, Sweden; 2 Department of Biosciences and Nutrition, 27106 Karolinska Institutet, Sweden; 3 Clinical Nutrition, Women’s Health and Allied Health Professionals, 59562 Karolinska University Hospital, Sweden; 4 Inflammation and Aging, Nursing Unit Aging, 59562 Karolinska University Hospital, Sweden; 5 Research and Development Unit, 83294 Stockholms Sjukhem, Sweden; 6 Department of Neurobiology, Care Science and Society, Division of Nursing, 27106 Karolinska Institutet, Sweden

**Keywords:** documentation, electronic health records, communication, dietetics, implementation science, terminology as topic

## Abstract

**Background:**

Standardised terminologies and classification systems play an increasingly important role in the continuous work towards high quality patient care. Currently, a standardised terminology for nutrition care, the Nutrition Care Process (NCP) Terminology (NCPT), is being implemented across the world, with terms for four steps: Nutrition Assessment (NA), Nutrition Diagnosis (ND), Nutrition Intervention (NI) and Nutrition Monitoring and Evaluation (NME).

**Objective:**

To explore associations between individual and contextual factors and implementation of a standardised NCPT among Swedish dietitians.

**Method:**

A survey was completed by 226 dietitians, focussing on: (a) NCPT implementation level; (b) individual factors; and (c) contextual factors. Associations between these factors were explored through a two-block logistic regression analysis.

**Results:**

Contextual factors such as intention from management to implement the NCPT (OR (odds ratio) ND 15.0, 95% Confidence Interval (CI) 3.9–57.4, NME 3.7, 95% CI 1.1–13.0) and electronic health record (EHR) headings from the NCPT (OR NI 3.6, 95% CI 1.4–10.7, NME 3.8, 95% CI 1.1–11.5) were associated with higher implementation. A positive attitude towards the NCPT (model 1 OR ND 3.8, 95% CI 1.5–9.8, model 2 OR ND 5.0, 95% CI 1.4–17.8) was also associated with higher implementation, while other individual factors showed less association.

**Conclusion:**

Contextual factors such as intention from management, EHR structure, and pre-defined terms and headings are key to implementation of a standardised terminology for nutrition and dietetic care.

**Implications for practice:**

Clinical leadership and technological solutions should be considered key areas in future NCPT implementation strategies.

## Introduction

A consistent use of terms and standards is a basic precondition for exchange of information within healthcare (Erickson et al., 2003; World Health Organization Executive Board, 2006). Documentation in electronic health records (EHR) needs to be clear and unambiguous, with a structured format, facilitating information extraction and interpretation for healthcare professionals from different professions and organisations, or with different roles in the healthcare system (Pain et al., 2017; Swan et al., 2019). Therefore, standardised terminologies and classification systems play an increasingly important role in the continuous work to improve health care to ensure delivery of high-quality patient care (McLachlan et al., 2020; World Health Organization Executive Board, 2006; Fennelly et al., 2021). Besides being an essential information communication channel for healthcare professionals, standardised clinical EHR documentation constitutes a great potential for outcomes management and evaluation of health care interventions, as well as for computerised clinical decision support systems (Ahmadian et al., 2011; Kim et al., 2020; McLachlan et al., 2020; Macieira et al., 2019; Lewis et al., 2021). To evaluate clinical data, compare outcomes, and merge data from different settings in a systematised way, terms must be defined and used unambiguously in the EHR (Oemig and Blobel, 2010). To respond to these needs, several terminologies and classification systems for health care were developed and implemented during recent decades, targeting both profession specific and interdisciplinary needs. Examples of terminologies providing terms for a certain discipline are the North American Nursing Diagnosis Association (NANDA) International nursing diagnoses (NANDA International, 2007) and the International Classification for Nursing Practice (INCP) (International Council of Nurses, 2021). Interprofessional terminologies or classification systems, such as the Systematised Nomenclature of Medicine Clinical Terms (SNOMED CT) (SNOMED CT, 2021) and the Logical Observation Identifier Names and Codes (LOINC) (Forrey et al., 1996), are developed to provide interdisciplinary connections between profession specific terminologies (Westra et al., 2008).

In nutrition care, attempts have been made previously to harmonise terms, mainly regarding nutrition-related medical diagnoses, such as malnutrition and obesity (Cederholm et al., 2019; Håkonsen et al., 2020; Phillips et al., 2020; World Health Organization, 2000). A comprehensive terminology for nutrition care provided by dietitians, was launched in 2008 by the American Academy of Nutrition and Dietetics (formerly the American Dietetic Association) (Swan et al., 2017, 2019; Thompson et al., 2015). The Nutrition Care Process Terminology (NCPT), builds upon a standardised model for nutrition care, the Nutrition Care Process (NCP). Nutrition Care Process Terminology contains defined terms for each of the four steps: Nutrition Assessment, Nutrition Diagnosis, Nutrition Intervention, and Nutrition Monitoring and Evaluation. During the last decade, the terminology has been translated into 12 languages (including Swedish), and terms for all four NCP steps have been implemented by dietitians across the world (Lövestam et al., 2019a). Most of these efforts have focussed on the implementation of step 2: Nutrition Diagnosis (Hakel-Smith, 2006; Lövestam et al., 2019a; Vivanti et al., 2017; Lewis et al., 2021). The NCPT is regularly updated and is currently being harmonised with and integrated into large international classification systems such as the SNOMED CT and the LOINC (SNOMED International, 2021; Academy of Nutrition and Dietetics, 2021). Templates from the NCPT are used in EHR systems across the world (Academy of Nutrition and Dietetics, 2021).

Implementation of health information innovations such as new terminologies is a long-term process, with several aspects affecting the implementation (Patiraki et al., 2017; Vivanti et al., 2017, 2018; Rajabpoor et al., 2018). Contextual aspects have been shown to be crucial (Cresswell and Sheikh, 2013; Harvey and Kitson, 2016; McCormack et al., 2002). The implementation of standardised diagnostic and classification systems among healthcare professionals have been associated with organisational aspects such as leadership, learning culture, and resources in terms of knowledge and time, and other aspects such as the percieved value of and need for the new system (Lee et al., 2013; Rajabpoor et al., 2018; Lövestam et al., 2017; Højen et al., 2014; Vivanti et al., 2017; Fennelly et al., 2021). The Integrated Promoting Action on Research Implementation in Health Services (i-PARIHS) framework was developed to identify essential aspects in implementation processes in health care (Harvey and Kitson, 2016; Rycroft-Malone, 2004). A successful implementation is explained by the interplay between four different main constructs: innovation, recipients, context and facilitation. Although innovation refers to the object of implementation, recipients are the people involved in the implementation, including their motivations, values and beliefs, as well as workplace resources and networks. The context construct focuses on three levels: local level (e.g. leadership and learning environment), organisational level (e.g. structure and organisational priorities) and external health system level (e.g. regulatory frameworks and inter-organisational networks). Facilitation includes the role of a person, either external or internal, supporting the implementation of changes in practice (Harvey and Kitson, 2016). Technological aspects such as compatibility with existing terminologies or EHR systems have been highlighted as key to implementation of standards in healthcare documentation (Vetter and Kim, 2021; Lee et al., 2013; Bennett and Steen, 2010; Santoro et al., 2021; Fennelly et al., 2021; Højen et al., 2014; Liu et al., 2010). Further, access to the EHR has been pointed out as a possible enabler for NCPT implementation among dietitians, both in an Australian study from 2013 and in a global survey from 2020 (O'Sullivan, 2013; Lövestam et al., 2020).

Implementation of e-health innovations is prioritised in the Swedish healthcare system, with the EHR as a standard in all healthcare settings for more than a decade (Jerlvall and Pehrsson, 2019; Swedish eHealth Agency, 2021). Efforts have been made to develop EHR integrated clinical decision support systems as well as outcomes management systems (Jerlvall and Pehrsson, 2019). To this end, the use of standardised terminologies has been prioritised, which is one reason why Swedish dietitians were early adopters of the NCPT, having started the implementation in 2010. Currently, Swedish dietitians have implemented the NCPT to a lesser extent when compared to the United States of America, Canada, and Australia, for example, but to a higher extent when compared to other non-English speaking countries such as Denmark, Norway, and Greece (Lövestam et al., 2019a). In order to enhance the use of standardised terminologies and classification systems in health care, we need to obtain knowledge regarding the individual and contextual factors associated with their implementation. Therefore, this study aimed to explore the associations between individual and contextual factors and implementation of standardised NCPT terms for each of the four NCP steps among Swedish dietitians.

## Method

### Design and survey

The International Nutrition Care Process and Terminology Survey (INIS) was developed and validated in 2016 in a multinational effort, involving 10 countries and seven languages, coordinated by the authors of this article (Lövestam et al., 2019b). The survey targeted NCP and NCPT implementation and attitudes among dietitians. Parts of this survey were combined with a newly developed Swedish questionnaire focusing on contextual factors. The parts of the INIS survey included:

• Implementation level of NCPT terms for each of the four NCP steps: Nutrition Assessment, Nutrition Diagnosis, Nutrition Intervention, and Nutrition Monitoring and Evaluation (usage rated on a 5-point Likert scale, where 1=*never*, 2=*rarely*, 3=*occasionally*, 4=*often* and 5=*always).*
• Individual factors (listed in Table 1)
• NCPT-related attitudes (16 statements to *agree* or *disagree* with on a 5-point Likert scale).

**Table 1.** Categorisation of response options regarding individual and contextual factors and implementation of a Nutrition Care Process (NCP) Terminology (NCPT).

Variables	Question	Categorisation of response options
DEPENDENT		
NCPT nutrition assessment implementation	To what extent do you use the standardised NCPT (the terminology, or the standardised terms) in your dietetic practice…	Low (never/rarely/occasionally) vs high (often/always)
	a) …For nutrition assessment documentation	
NCPT nutrition diagnosis implementation	b) …For nutrition problem (nutrition diagnosis) documentation	Low (never/rarely/occasionally) vs high (often/always)
NCPT nutrition intervention implementation	c) …For nutrition intervention documentation	Low (never/rarely/occasionally) vs high (often/always)
NCPT nutrition monitoring and evaluation implementation	d) …For documentation of monitoring and evaluation of nutrition care	Low (never/rarely/occasionally) vs high (often/always)

INDEPENDENT		
**Individual factors**		
Education level	What is the highest level of education you have completed in nutrition or dietetics?	Higher (master, doctoral) vs lower (Bachelor)
Graduated from dietetic education	What year did you complete your training to be able to practice as a dietitian?	Recently (≤5 years ago) vs longer ago (>5 years ago)
Learnt NCPT as student	Where did you learn about NCP/NCPT? Please select all that apply	Yes (selected response option ‘as dietetic student/intern’) vs no (did not select response option ‘as dietetic student/intern’)
NCPT attitude	Please select your agreement with the following statements regarding the NCP and NCPT (20 statements)	Positive (>50% agree) vs negative (≤50% agree)

**Contextual factors**		
*Workplace culture*		
Have colleagues	Do you work in an organisation where you have one or more dietetic colleagues?	Yes (yes or partly) vs no (no)
Colleagues use the NCPT	Do you mainly work in a setting where one or more of your colleagues use the NCP/NCPT?	Yes (yes) vs no (no/don’t know)
NCPT discussions at workplace	How often are NCP/NCPT discussed at your workplace in an organised way (e.g. workplace meetings or education sessions)	Rarely (Once every 6 months or less) vs often (more than 1 time every 6 months)
NCPT discussions at network meetings	How often do you participate in sessions outside your main workplace, where NCP/NCPT are being discussed (e.g. regional dietetic meetings, education sessions)?	Rarely (Once every 6 months or less) vs often (more than 1 time every 6 months)
NCPT discussions online	How often do you participate in discussions about NCP/NCPT at email lists or in social media?	Rarely (Once every 6 months or less) vs often (more than 1 time every 6 months)
*Management*		
Manager is a dietitian	Is one or more of your manager(s) a registered dietitian?	Yes (yes) vs no (no, don’t know)
Intention from management	To what extent do you agree with the following statement: At my workplace, management clearly want dietitians to use NCP/NCPT	Yes (agree/strongly agree) vs no (strongly disagree/disagree/neutral)
NCPT knowledgeable management	To what extent do you agree with the following statement: I Percieve that my manager has knowledge about NCP/NCPT	Yes (agree/strongly agree) vs no (strongly disagree/disagree/neutral)
*Workplace resources*		
EHR template follows NCP	Is your EHR structured in a way that supports the four NCP steps?	Yes (yes/partly) vs no (no/don’t know)
EHR headings from NCPT	Are the headings in your EHR based on the NCP terminology?	Yes (yes/partly) vs no (no/don’t know)
Nutrition care plans follow NCPT	Are the nutrition care plans used at your workplaced adjusted for NCP/NCPT use?	Yes (yes/partly) vs no (no/we don’t use routines/don’t know)
*Feedback processes*		
Goal setting	At your workplace, has there been any goal set for the NCP/NCPT implementation (e.g. proportion of PES in dietetic clinical documentation)?	Yes (yes, currently/yes, recently) vs no (no/don’t know)
*Access to facilitators*		
Internal facilitator	Are/were there one or more persons at your workplace which have been assigned a facilitating role in the NCP/NCPT implementation?	Yes (yes) vs no (no, but had earlier/no, but plan to have/no/don’t know)
External facilitator	Are there one or more external persons (consultants, educators etc), with a facilitating role in the NCP/NCPT implementation at your workplace?	Yes (yes) vs no (no, but had earlier/no, but plan to have/no/don’t know)

The Swedish questionnaire was developed in 2016, guided by the iPARIHS framework (Harvey and Kitson, 2016), to be able to assess contextual factors that might affect NCPT implementation. The questionnaire was tested regarding content validity and face validity, resulting in a Scale Content Validity Index of 0.95 (Polit and Beck, 2006). Questions are listed in Table 1.

### Data collection procedure

Inclusion criteria for the study was being a registered dietitian in Sweden, which included dietitians working in clinical settings as well as other settings such as food service. The survey, including both the INIS survey and the new Swedish questionnaire, was disseminated online in February–April 2017, through SurveyMonkey, and a link to this webpage was sent to Swedish dietitians (*n*∼1200) through email newsletters, Facebook groups, and emailing lists for Swedish dietitians, together with an invitation letter, containing information about the study. Reminders were sent out twice: after threeweeks and after sixweeks. To be able to enter the survey, all participants needed to provide their consent electronically.

### Data analyses

Descriptive statistics were used to identify the characteristics and frequency of different individual and contextual factors. The categorisation of factors as individual or contextual is listed in Table 1.

Table 1 shows all variables and how the responses were categorised for the following analyses. Four dependent variables were used: implementation of standardised terminology regarding (i) Nutrition Assessment, (ii) Nutrition Diagnosis, (iii) Nutrition Intervention, and (iv) Monitoring and Evaluation. For each of these four variables, the reported usage was categorised as *low* or *high* (Table 1). The 16 questions regarding NCP and NCPT attitudes were grouped into one variable, in the following way: For each of the 16 statements, respondents were asked to *agree* or *disagree*, on a scale between 1=*totally disagree* and 5=*totally agree*. Percentages were calculated for the statements where respondents stated *agree* (4) or *totally agree* (5), and those agreeing with >50% of the statements were categorised as having a positive attitude toward NCPT. A more detailed analysis of the attitudes questions has been published elsewhere (Lövestam et al., 2020). Correlations between variables were assessed using a three-phase process:

1) Bivariate analyses (Spearman’s *ρ*) examined the correlations between the four NCPT implementation variables (dependent variables) and the independent variables, which were grouped in clusters representing individual factors and contextual factors.
2) Variables were selected for inclusion in the logistic regression analysis. All variables categorised as individual factors were included. For the contextual factors, the results from the bivariate analysis determined whether the variable was included or excluded from the regression analysis. Variables showing significant correlations (p<0.05) with implementation of standardised terms for at least one of the four NCP steps were selected to be included in the logistic regression analysis. Two variables were excluded at this stage due to multicollinearity (‘Have colleagues’ and ‘EHR template follows NCP’).
3) Logistic regression analysis was performed with the implementation of standardised NCPT terms for each of the four NCP steps as dependent variables. The logistic regression was performed in two blocks, where the first block contained the individual factors, and the second block contained both individual and contextual factors. Odds ratio (OR) and 95% confidence intervals (CI) were calculated and evaluated with significance tests.

### Ethics approval

Ethics approval was granted by the Regional Ethical Board in Uppsala (Dnr 2016/258).

## Results

In total, 274 persons completed the survey. After excluding non-dietitians (*n*=5) and dietitians not working with patient-related tasks *(n*=43), 226 respondents were included in the analysis. High usage of the NCPT (*often* or *always*) was reported by 22% (Nutrition Assessment), 51% (Nutrition Diagnosis), 22% (Nutrition Intervention) and 18% (Nutrition Monitoring and Evaluation), respectively.

### Individual factors

A majority of the respondents had graduated between 6 and15 years ago, with a Bachelor’s degree or equivalent as the most common educational level. About one-third graduated 5 years ago or more recently. A large majority (86%) were classified as having positive attitudes toward the NCPT (Table 2). A positive attitude (OR 5.0, 95% CI 1.4–17.8) and higher education (OR 3.0, 95% CI 1.0–8.7) were associated with a higher implementation of Nutrition Diagnosis terminology (Table 3). Those who graduated from dietetic training earlier than 5 years ago were associated with a lower Nutrition Assessment (OR 0.2, 95% CI 0.0–1.0) and Nutrition Monitoring and Evaluation (OR 0.2, 95% CI 0.0–0.9) terminology usage compared to those who more recently finished their training to be dietitians (Table 3).

**Table 2.** Occurrence of individual and contextual factors and their correlation (Spearman’s ρ) to implementation of a 4-step Nutrition Care Process Terminology (NCPT).

		Step 1: Nutrition assessment		Step 2: Nutrition diagnosis		Step 3: Nutrition intervention		Step 4: Nutrition monitoring and evaluation	
	Occurrence (%)	Correlation coefficient	p	Correlation coefficient	p	Correlation coefficient	p	Correlation coefficient	p
Individual factors									
Education level >bachelor degree	28	−0.036	0.596	0.101	0.134	−0.036	0.596	0.020	0.763
Graduated from dietetic education >5 years ago	72	−0.129	0.056	0.047	0.486	−0.136	0.042	−0.152	0.023
Learnt NCPT as student	38	0.026	0.703	−0.074	0.275	0.066	0.326	0.087	0.196
Positive NCPT attitude	86	0.110	0.103	0.211	0.002	0.074	0.274	0.043	0.528
Contextual factors									
Workplace culture									
Have colleagues^a^	81	−0.048	0.477	0.210	0.002	0.030	0.654	0.047	0.487
Colleagues use NCPT	71	0.084	0.265	0.464	0.000	0.158	0.034	0.209	0.005
NCPT discussions at workplace	31	0.069	0.307	0.327	0.000	0.124	0.065	0.154	0.022
NCPT discussions at network meetings^b^	7	0.029	0.666	0.048	0.479	−0.055	0.417	0.013	0.844
NCPT discussions online^b^	9	0.016	0.808	0.070	0.301	−0.018	0.795	−0.029	0.673
Management									
Manager is a dietitian	32	−0.049	0.469	0.240	0.000	0.005	0.941	0.021	0.754
Intention from management	41	0.042	0.531	0.481	0.000	0.141	0.036	0.157	0.019
NCPT knowledgeable management	31	−0.047	0.483	0.264	0.000	0.031	0.648	0.044	0.510
Workplace resources									
EHR template follows NCP^a^	83	0.117	0.081	0.259	0.000	0.113	0.092	0.080	0.236
EHR headings from NCPT	58	0.206	0.002	0.309	0.000	0.235	0.000	0.204	0.002
Nutrition care plans follow NCPT	44	0.178	0.008	0.392	0.000	0.167	0.013	0.145	0.030
Feedback processes									
Goal setting	39	−0.027	0.692	0.338	0.000	0.027	0.693	0.080	0.236
Access to facilitators									
Internal facilitator	34	−0.012	0.863	0.212	0.002	0.089	0.186	0.115	0.089
External facilitator^b^	6	0.005	0.943	0.092	0.175	−0.039	0.565	−0.018	0.789

^a^Excluded from regression analysis due to multicollinearity.

^b^Excluded from regression analysis due to non-significant correlations (p>0.05).

**Table 3.** 2-block logistic regression analysis: odds ratio for higher implementation of the 4-step Nutrition Care Process Terminology (NCPT) in associated variables categorised as individual or contextual factors.

	Step 1: Nutrition assessment				Step 2: Nutrition diagnosis				Step 3: Nutrition intervention				Step 4: Nutrition monitoring and evaluation			
	Model 1 (95% CI)	χ2	Model 2 (95% CI)	χ2	Model 1 (95% CI)	χ2	Model 2 (95% CI)	χ2	Model 1 (95% CI)	χ2	Model 2 (95% CI)	χ2	Model 1 (95% CI)	χ2	Model 2 (95% CI)	χ2
**Block 1: Individual factors**				7.3				10.5*				5.1				6.2
Education level >bachelor degree	1.2 (0.5–3.0)		1.5 (0.6–4.1)		1.5 (0.7–3.1)		3.0* (1.0–8.7)		0.8 (0.3–2.0)		0.8 (0.3–2.3)		1.4 (0.6–3.6)		1.7 (0.6–5.0)	
Graduated from dietetic education >5 years ago	0.2* (0.0–1.0)		0.2 (0.0–1.1)		1.0 (0.3–3.0)		0.7 (0.1–3.0)		0.4 (0.1–1.5)		0.3 (0.1–1.4)		0.3 (0.1–1.4)		0.2* (0.0–0.9)	
Learnt NCPT as student	0.4 (0.1–2.0)		0.5 (0.1–2.3)		0.8 (0.3–2.0)		0.7 (0.2–2.5)		0.7 (0.2–2.8)		0.8 (0.2–3.3)		0.9 (0.2–3.7)		1.1 (0.2–4.9)	
Positive NCPT attitude	2.0 (0.6–7.4)		1.6 (0.4–6.9)		3.8* (1.5–9.8)		5.0* (1.4–17.8)		1.3 (0.4–4.2)		0.8 (0.2–2.9)		1.1 (0.3–3.6)		0.5 (0.1–2.0)	
**Block 2: Contextual factors**				24.8*				94.0**				26.8*				33.7**
*Workplace culture*																
Colleagues use NCPT			1.4 (0.5–4.1)				6.3*** (2.2–17.7)				1.8 (0.6–5.7)				3.0 (0.7–13.5)	
NCPT discussions at workplace			1.3 (0.5–3.1)				1.7 (0.7–4.4)				1.2 (0.5–2.8)				1.7 (0.7–4.3)	
*Management*																
Manager is a dietitian			0.4 (0.1–1.6)				0.6 (0.1–2.3)				0.2* (0.1–0.9)				0.2 * (0.0–0.8)	
Intention from management			1.8 (0.6–5.6)				15.0*** (3.9–57.4)				2.7 (0.9–8.6)				3.7* (1.1–13.0)	
NCPT knowledgeable management			0.6 (0.2–2.0)				0.3 (0.1–1.2)				0.8 (0.2–2.5)				0.7 (0.2–2.3)	
*Workplace resources*																
EHR headings from NCPT			2.5 (0.9–6.7)				1.3 (0.6–3.0)				3.8* (1.4–10.7)				3.6* (1.1–11.5)	
Nutrition care plans follow NCPT			3.0* (1.2–7.4)				5.0*** (2.0–13.0)				1.7 (0.7–4.1)				1.6 (0.6–4.4)	
*Feedback processes*																
Goal setting			0.6 (0.2–1.7)				1.9 (0.7–5.4)				0.7 (0.3–2.0)				1.2 (0.4–3.4)	
*Access to facilitators*																
Internal facilitator			1.4 (0.5–4.0)				0.2* (0.1–0.7)				2.1 (0.7–6.2)				2.0 (0.6–6.2)	
Final model		7.3		32.1*		10.5*		104.5**		5.1		31.9*		6.2		39.9**

*p<0.05.

**p<0.01.

***p<0.001.

### Contextual factors

Occurrence of contextual factors together with their correlation with implementation of standardised NCPT terms for each of the four NCP steps are presented in Table 2. Workplace culture, represented by the item *Colleagues use the NCPT* stood out as a predictor of Nutrition Diagnosis terminology implementation (Table 3, OR 6.3, 95% CI 2.2–17.7), while no such association was seen with the other three NCP steps in the regression analysis. However, in the bivariate analyses (Table 2), positive associations were identified between *Colleagues use the NCPT* and implementation of Nutrition Intervention (*ρ* 0.158, *p* = 0.034) and Nutrition Monitoring and Evaluation (*ρ* 0.209, *p* = 0.005) terminology. Furthermore, *NCPT discussions at workplace* was positively associated with implementation of Nutrition Diagnosis (*ρ* 0.327, *p*<0.001) and Nutrition Monitoring and Evaluation (*ρ* 0.154, *p*=0.022) terminology (Table 2).

An association was seen between the agreement with the statement that there is an *intention from management* to implement the NCPT and a higher probability to use both Nutrition Diagnosis (OR 15.0, 95% CI 3.9–57.4) and Nutrition Intervention (OR 3.7, 95% CI 1.1–13.0) terminology (Table 3). For those who stated that at least one of the managers at their workplace was a dietitian, the bivariate analysis showed a positive correlation (*ρ* 0.240, *p*<0.001) to Nutrition Diagnosis terminology implementation (Table 2). However, this correlation was not confirmed in the regression analysis, where a negative association was found with Nutrition Intervention (OR 0.2, 95% CI 0.1–0.9) and Nutrition Monitoring and Evaluation (OR 0.2 95% CI 0.0–0.8) terminology implementation (Table 3).

*Electronic health record headings from NCPT* was associated with a higher implementation of Nutrition Intervention (OR 3.8, 95% CI 1.4–10.7) and Nutrition Evaluation and Monitoring (OR 3.6, 95% CI 1.1–11.5) terminology (Table 3). Further, in the bivariate analysis, *EHR headings from NCPT* was associated with Nutrition Assessment terminology implementation (*ρ* 0.206, *p*=002), and Nutrition Diagnosis implementation was associated with both the *EHR template follows NCP* (*ρ* 0.259, *p*<0.001) and *EHR headings from NCPT* (*ρ* 0.309, *p*<0.001).

Having *Nutrition care plans [that] follow NCPT* was associated with a higher probability of Nutrition Assessment (OR 3.0, 95% CI 1.2–7.4) and Nutrition Diagnosis (OR 5.0, 95% CI 2.0–13.0) terminology implementation (Table 3). In the bivariate analysis, Nutrition Intervention (*ρ* 0.167, *p*=0.013) and Nutrition Monitoring and Evaluation (*ρ* 0.145, *p*=0.030) terminology use were also associated with *Nutrition care plans [that] follow NCPT* (Table 2).

In the bivariate analysis, a correlation was found (*ρ* 0.338, *p*<0.001) between *goal setting* and Nutrition Diagnosis terminology implementation, but not confirmed in the regression analysis (Table 3). Although the bivariate analysis showed a positive association between access to *Internal facilitator* and Nutrition Diagnosis terminology implementation (*ρ* 0.212, *p* = 0.212), the logistic regression showed a negative association between these factors (OR 0.2, 95% CI 0.1–0.7).

## Discussion

Advantages of an adequately used standardised terminology among healthcare professionals are numerous, regarding patient safety, outcomes management and research opportunities (Erickson et al., 2003; Ahmadian et al., 2011; McLachlan et al., 2020; Kim et al., 2020; Oemig and Blobel, 2010; Lewis et al., 2021). However, implementation is a challenge, as evident from the findings from this survey, where the standardised NCPT terms for the different NCP steps were used by 18–51% of the participants. This is not unique for the nutrition and dietetic specific terminology, as efforts to implement standardised healthcare terminologies and classification systems into clinical practice have faced several obstacles during the last few decades (Kim et al., 2020; Lee et al., 2013; Liu et al., 2010; Fennelly et al., 2021; Højen et al., 2014). Results of this study illustrate the complex interplay between the individual and contextual factors that affect this process.

Nutrition Care Process Terminology terms for Nutrition Diagnosis stand out from the other steps in NCP with regard to both the implementation level and connection to implementation enablers. Individual factors such as positive attitudes, as well as contextual factors such as having colleagues using the NCPT, and clear implementation intention from the management, seem to have a stronger association with implementation of NCPT for Nutrition Diagnosis compared to the other steps. One reason for this emphasis on the Nutrition Diagnosis might be that individuals, colleagues and management perceive this step as more useful, compared to the other steps. Both internationally and in a Swedish context, the NCPT for Nutrition Diagnosis has been more explicitly promoted, compared to the other three steps, in implementation efforts as well as in research and education (Hakel-Smith, 2006; Lövestam et al., 2017; Lewis et al., 2021; Vivanti et al., 2017). The question remains whether dietitians value the Nutrition Diagnosis terminology higher, when compared with the other NCP steps, because they have received more training in recognising the benefits of this step. An educational approach integrating all steps in the nursing process instead of teaching them separately has been suggested in nursing (Patiraki et al., 2017). Using an integrative educational approach might also help dietitians to see the value of using the standardised NCPT terms for all four NCP steps, for example, by highlighting the links between the four steps and how they inform each other (Lewis et al., 2021; Thompson et al., 2015).

In the i-PARIHS framework, the ‘recipient’ construct represents the people who are affected by and influence the implementation of a certain innovation. The individual factors included in this survey, such as participants’ educational level or whether they learnt NCPT as students, explained the variations in the implementation level only to a small extent. Some differences between groups were identified. The i-PARIHS recipient construct considers knowledge and skills as an important aspect affecting implementation (Harvey and Kitson, 2016). One probable reason that the more recently educated dietitians had a higher NCPT usage is the recency of their NCPT information and training. The aspect of knowledge and skills is, however, more complex than merely concerning knowledge levels regarding the NCPT. It also involves the perspective that more experienced dietitians might already have developed a structure that they perceive as well-functioning for their professional assessment and documentation in the EHR, while more recently educated dietitians need to be more open to new frameworks and other innovations intended to support them as professionals. The level of usability of an innovation is highlighted as a major aspect in the implementation process in the i-PARIHS framework (Harvey and Kitson, 2016), and more recently educated dietitians might thus see the NCPT as a more useful innovation for their clinical documentation compared to the more experienced professionals.

The EHR environment is a resource that can be connected to both the ‘recipient’ and the ‘context’ constructs in the i-PARIHS framework (Harvey and Kitson, 2016). In an Australian study from 2013, dietitians reported increased confidence in NCPT use if they had access to an electronic documentation system (O'Sullivan, 2013). Simplicity in the process of entering data into the EHR has been pointed out as a key factor for overall documentation quality (Vetter and Kim, 2021) as well as the implementation of standardised terms in, for example, SNOMED CT (Lee et al., 2013; Bennett and Steen, 2010; Fennelly et al., 2021). For two NCP steps: Nutrition Intervention, and Nutrition Monitoring and Evaluation, this was confirmed through an association between the existence of pre-set EHR pre-defined headings and the implementation level of standardised NCPT terms. Structured templates in the EHR have been shown to improve quality of both the clinical documentation and the care given (Santoro et al., 2021; Vetter and Kim, 2021). The design of intuitive data entry interfaces, such as autocomplete functions, abbreviation and acronym expansion (Lee et al., 2013), or check boxes and drop-down lists (Bennett and Steen, 2010), have been suggested as important implementation strategies for the SNOMED CT terms. It is likely that this also is applicable to the NCPT, especially with regards to the ongoing harmonisation between NCPT and SNOMED CT (SNOMED International, 2021). With health professionals taking part in the future development of EHR systems and related templates, allowing for further simplicity in data entering, this might result in increased usage of standardised terminologies such as NCPT.

Intention from management showed a very strong association with higher NCPT implementation levels, confirming the important role of leadership in an implementation process, as emphasised in earlier studies (Harvey and Kitson, 2016; Vivanti et al., 2017; Lövestam et al., 2017). Therefore, it is interesting that less than half of all participants agreed that their management clearly wanted dietitians to use the NCPT. In the i-PARIHS framework (Harvey and Kitson, 2016), successful leadership was described as an inclusive and transformational leadership, resulting in clear roles, effective teamwork, and effective organisational structures (Rycroft-Malone, 2004; McCormack et al., 2002; Harvey and Kitson, 2016). Educational efforts targeting clinical leaders, focussing on general leadership skills as well as the role and purpose of standardised terminologies, might thus be an important part in a future implementation strategy for NCPT as well as other healthcare terminologies.

### Limitations of the study

Data in this study were collected in 2017, and with the rapid development of new EHRs and other information systems in health care, some circumstances from the study setting might already have changed, causing changes in NCPT usage among dietitians in Sweden. About one-fourth of all Swedish dietitians participated in this survey. A larger sample might have resulted in a regression model showing more robust results. There are several possible reasons for not achieving a higher response rate. Firstly, persons with a registration as dietitian and who work in non-patient related settings might have viewed this survey as irrelevant for them to complete. Secondly, the dissemination strategy relied on newsletters, emailing lists and social media groups that might only have reached parts of the population. The dissemination strategy did not allow a non-response analysis, but it can be assumed that those participating in the survey were those who were more interested in the NCPT and that the results therefore are not generalisable to all Swedish dietitians. Notwithstanding this, patterns identified in this study are interesting to highlight and to discuss in relation to earlier studies, implementation theories and societal phenomena, as they can provide valuable perspectives and insights for future research, implementation initiatives and strategies related to dietitians’ use of standardised terminology. Bivariate and regression analysis were combined in this study, sometimes resulting in contradicting results. In these cases, the regression analysis should be considered the more robust method. Nonetheless, results from the bivariate analyses presented in this article allow the reader to follow the selection of variables for the regression analysis and to be able to see and interpret the results from both analyses. The two-step analysis performed in this study required multiple statistical analyses, which increase the risk of false positive results. The results regarding both the bivatiate and regression analyses should therefore be interpreted with caution. It should also be considered that four separate outcome variables were used in the analyses: implementation of standardised NCPT terms for Nutrition assessment, Nutrition Diagnosis, Nutrition Intervention and Nutrition Monitoring and Evaluation. Adjusted *p*-values or correction for multiple testing was not used; instead, *p*-values have been presented in detail (Table 2) or at three levels (Table 3) to allow the reader to interpret the results.

## Conclusion

Based on the results of this survey, we suggest that contextual factors should be in focus for a strategy to implement the NCPT, with the management level as a key area for successful implementation. Electronic health record structure and predefined headings should not be underestimated in the development of implementation strategies, and we further suggest that future research should focus on the effect of technological solutions to facilitate the entry of standardised terms into the EHR.
